# Stem injection of ^15^N–NH_4_NO_3_ into mature Sitka spruce (*Picea sitchensis*)

**DOI:** 10.1093/treephys/tpu084

**Published:** 2014-10-20

**Authors:** Richard Nair, Andrew Weatherall, Mike Perks, Maurizio Mencuccini

**Affiliations:** 1 School of Geosciences, University of Edinburgh, Crew Building, West Mains Road, Edinburgh EH9 3JN, Midlothian, Scotland; 2 National School of Forestry, University of Cumbria, Penrith, CA11 0AH, UK; 3 Northern Research Station, Forest Research, Roslin EH25 9SY, Midlothian, Scotland; 4 ICREA at CREAF, Campus de UAB, Cerdanyola del Valle's, Bellaterra, Barcelona, Spain

**Keywords:** canopy position, isotope labelling, labelled biomass, nitrogen allocation, nitrogen storage, xylem

## Abstract

Stem injection techniques can be used to introduce ^15^N into trees to overcome a low variation in natural abundance and label biomass with a distinct ^15^N signature, but have tended to target small and young trees, of a variety of species, with little replication. We injected 98 atom% ^15^N ammonium nitrate (NH_4_NO_3_) solution into 13 mature, 9- to 13-m tall edge-profile Sitka spruce trees in order to produce a large quantity of labelled litter, examining the distribution of the isotope throughout the canopy after felling in terms of both total abundance of ^15^N and relative distribution of the isotope throughout individual trees. Using a simple mass balance of the canopy alone, based on observed total needle biomass and modelled branch biomass, all of the isotope injected was accounted for, evenly split between needles and branches, but with a high degree of variability both within individual trees, and among trees. Both ^15^N abundance and relative within-canopy distribution were biased towards the upper and middle crown in foliage. Recovery of the label in branches was much more variable than in needles, possibly due to differences in nitrogen allocation for both growth and storage, which differ seasonally between foliage and woody biomass.

## Introduction

Interest in the role of the nitrogen (N) cycle in ongoing global change has driven a large number of studies into the effects of N deposition and the dynamics of N pools within ecosystems (e.g., Nadelhoffer et al. 1999*b*, Magill et al. 2004, Magnani et al. 2007). Nitrogen's stable isotope, ^15^N, is often used as an enriched tracer in spikes of mineral ^15^N additions (e.g., Högberg 1997, Nadelhoffer et al. 1999*a*, Mulholland and Tank 2000, Templer et al. 2012), or at natural abundance (e.g., Högberg 1990, Dijkstra et al. 2008), to investigate N dynamics beyond that which can be measured in bulk changes in pools and fluxes. However, as ecosystem δ^15^N is typically highly conserved (Robinson 2001), soil δ^15^N is spatially variable (Högberg 1997) and temperate decomposition rates are relatively slow (Vitousek and Howarth 1991), it is consequently very difficult to trace ^15^N from litter pools without a source of biomass with a δ^15^N high enough to allow detection. Labelled biomass must be even more enriched if short-term recovery of the label is desired, or if one intends to trace the label into relatively uncompetitive pools, with high dilution, such as trees (Nadelhoffer et al. 1999*b*).

Biomass enriched in ^15^N can be produced by application of labelled fertilizers (Weatherall et al. 2006, Langenbruch et al. 2013), foliar sprays (Zeller et al. 1998) or by direct injections into the plant vascular system (Swanston and Myrold 1998). This latter methodology is potentially most efficient as the valuable ^15^N-labelled material is not lost via misting (Bowden et al. 1989), exposed to soil sinks (Nadelhoffer et al. 1999*b*) or exported from the immediate area by soil hydrology. Injection techniques (Roach 1939) were first utilized to apply enriched ^15^N compounds by Horwath et al. (1992) and consist of a reservoir of injection substrate introduced into the tree either passively (Proe et al. 2000, Christenson et al. 2002, Garten and Brice 2009, Churchland et al. 2012) or under pressure (Horwath et al. 1992, Swanston and Myrold 1998), via a purpose-drilled hole accessing the cambium and plant vascular system where the solution is taken up via a Venturi effect. This method can be used to trace the fate of injected elements either within the trees (Horwath et al. 1992, Swanston and Myrold 1998, Augusto et al. 2011) or into the soil system (Garten and Brice 2009, Churchland et al. 2012), but it has rarely been used (Christenson et al. 2002, Weatherall 2005) as a method with the primary purpose of creating labelled biomass, typically targeting relatively young and small trees, where total biomass is low, and the canopy both open and easily sampled. It is difficult to draw conclusions about the overall effectiveness of this method because of the large variety of species employed (Table 1), but generally, it appears that in conifers, injected N is heterogenously distributed within tree crowns both in the short term (Augusto et al. 2011), and even more so as N is translocated throughout the canopy by the tree. These differences may be caused by within-canopy variation in N demand due to exposure and related photosynthetic activity (Ellsworth and Reich 1993), or variations in needle age and N storage potential (Proe and Millard 1994), which may vary in larger trees both due to allometric scaling of tree proportions (Niklas 1995), and the effects of canopy closure on crown size. Both of these changes also reduce the absolute amounts of foliage to woody biomass within the tree (Ritson and Sochacki 2003), which may also affect the fate of injected ^15^N between foliar and woody pools. Evergreen species also retain needles for several years (6–8 years in Sitka spruce (*Picea sitchensis* (Bong.) Carr.) Norman and Jarvis (1974)), so younger trees may not represent the full range of needle age classes present in older individuals.

**Table 1.** Summary of selected stem injection experiments using a ^15^N label. Studies have been included to demonstrate the variety of purposes, species and tree sizes employed.

Reference	Species	Habit	Purpose	Height (m)	*n*
Horwath et al. (1992)	*Populus* clone	Deciduous	Trace to roots/soil system	Not given	8 (2 × 4)
Swanston and Myrold (1998)	*Alnus rubra*	Deciduous	Partitioning within crown	5	10
Proe et al. (2000)	*Pinus radiata*	Evergreen	Partitioning within crown	5–6	8 (2 × 4)
Christenson et al. (2002)	*Quercus velutina*	Deciduous	Herbivorous moth frass	9	1
Weatherall (2005)	*Picea sitchensis*	Evergreen	Production of labelled biomass	3.2	7
Garten and Brice (2009)	*Liquidambar styraciflua*	Deciduous	Partitioning belowground	17	24 (2 × 3 × 2)
Augusto et al. (2011)	*Pinus pinaster*	Evergreen	Foliar labelling	4.8	3

As well as tree biomass size and proportions, the size of N pools within the tree and their sink strengths change over the growing season, both due to phenological variation in nutrient assignment (Weinstein et al. 1991) and overwinter storage of ^15^N in current-year needles and roots (e.g., Millard and Proe (1992) for Sitka spruce). In a study on 4-year-old *Pinus radiata* (D. Don), Proe et al. (2000) initially recovered 45% of the injected ^15^N in the canopy 1 week after injection, rising to 83% at the end of the growing season (8 months after injection), with a bias in ^15^N recovery away from the upper canopy, while in Sitka spruce saplings, the majority of an injected ^15^N–NH_4_NO_3_ solution was found in the above ground biomass of the harvested trees (Weatherall 2005).

The aim of this study was to produce a quantity of ^15^N enriched Sitka spruce (*P. sitchensis*) biomass suitable for a subsequent field study, requiring hundreds of kilograms of dry, isotope-labelled foliage for replacement of litter layers. As the intention was to produce as much labelled foliar biomass as possible, it was planned to inject trees on the edge of our target stand, because they were expected to have relatively more foliage than inside the closed canopy (Zavitkovski 1981). A potential consequence of this approach is that edge trees may display spatial variability in ^15^N recovery because of factors that affect intra-canopy ^15^N distribution. The trees ranged in heights from 9 to 13 m, and we investigated differences in ^15^N recovery and distribution in the canopy due to variations in tree and crown morphology.

## Materials and methods

### Site and stand characteristics

A 20-year-old stand comprising 90% Sitka spruce and 10% *Larix decidua* (European larch) was selected in Cardrona forest, a mixed conifer plantation forest in the Scottish Borders (55°61′50″N–3°12′87″E), ∼38 km south of Edinburgh. The site was a hillside with well draining, brown forest soil (annual rainfall of 887 mm and mean monthly temperatures between 0 and 18 °C). The stand was selected as it fulfilled the criteria of having a long, accessible stand edge (0.6 km) of (predicted by forest inventory GIS) 10–12-m tall Sitka spruce, close to a forest road, while not being located on any major recreational routes through the forest.

### Injection method

Stem injections were carried out in July and August 2011 with the trees remaining in the field until December 2011. Twenty-one trees with diameter at breast height (DBH) between 12 and 25 cm and no visible wounds or deformities at breast height (1.3 m) were prepared for the ^15^N injection along the stand edge.

Our injection apparatus was based on a passive uptake design (Proe et al. 2000). The apparatus consisted of a reservoir (an inverted 1 l bottle with two 10 mm holes in the raised base), affixed to the tree and connected by a 3-mm diameter tube to a 20-mm diameter, double-holed bung. A second 3-mm tube from the bung was closed with an adjustable plastic tap. The trees were prepared by removing an area of bark ∼1 m from the ground on the inside of the stand with sandpaper, and drilling a 35 (depth) × 20 (radius) mm hole using a hand drill with a wood auger bit. Once drilled, the hole was immediately plugged with the bung and coated on its sides and surface with a commercially available waterproof silicone sealant. For each tree, the reservoir was pre-filled with deionized (DI) water and allowed to flow through the apparatus by operating the tap, flooding the wound site and draining out, to refill the wound as quickly as possible and limit cavitation. Once air bubbles had been flushed from the system, the tap was closed, leaving 1 l empty volume in the reservoir, which was then filled with dilute red Safranin dye, and the tap was adjusted to bring the coloured solution to the injection site. The next day, trees without obvious uptake or with evidence of leaks (eight of the 21 trees prepared for ^15^N injections) were eliminated from the experiment. For the remaining trees, the apparatus was partially drained using the tap to leave 1 l of empty capacity, and filled with 1 l of the injection solution. The injection was 1 l of 21 g l^−1^ double-labelled 98 atom% ^15^NH_4_^15^NO_3_ (CK Gas Products, Hampshire, UK), delivering ∼7.53 g ^15^N or 0.3–0.8% of the total tree N pool, depending on the size of the tree. Ammonium nitrate was used for the injections as both of its constituent ions are transported in the xylem stream (Marschner and Marschner 2012), with a label equally distributed between the anion and the cation in case of differential assignment within the tree. After the introduction of the solution, the uptake (in ml) from the bottles was recorded from every reservoir every 1–2 days, and at each occasion the reservoir was refilled to 1 l by addition of further DI water to prevent the equipment running dry between refills, while steadily diluting the solution. A linear rate of uptake from the bottles was assumed and the bottles were topped up until the estimated NH_4_NO_3_ concentration in each bottle was <1 g l^−1^ in all bottles. The bottles were then allowed to run dry and stand for several days before deconstruction.

### Sampling strategy and analysis

All 13 trees were felled in December 2011, 4.5 months after the injection, along with an unlabelled tree as a control. All branches were immediately cut away from the main stem and bundled into six categories per tree, representing the specific location of removal along the main stem, in combinations of three vertical sections: Canopy_BOT_ (from the base of the tree to 3.5 m up the trunk), Canopy_MID_ (from 3.5 to 7.5 m up the trunk) and Canopy_TOP_ (from 7.5 m to the top of the tree) and two radial sections: Canopy_IN_ (comprising 120° inside the stand) and Canopy_OUT_ (comprising 240° facing out of the stand), with each of the six spatial positions having both a vertical (Canopy_TOP_, Canopy_MID_ or Canopy_BOT_) and radial (Canopy_IN_ or Canopy_OUT_) identifier. The bundles were either removed from the site immediately and transported to the location for further processing, 28 km away, or due to the large volume of biomass, left on the site for 3 weeks until early January 2012. Both sets of branches were stored outside away from sites where water would accumulate, under tarpaulin, until all had been collected in early January. During this period most precipitation at both sites was snow which had not substantially melted at the time of collection. Once all branches were collected, all the bundled sections were moved inside a dry polytunnel and chopped into small sections using a chainsaw and manual loppers. This material was then dried in batches in a timber kiln for up to 2 weeks at 70 °C, but, due to the time required per batch, around three-quarters of the material was found to be sufficiently dry to cause needle shedding after temperatures in the polytunnel reached 40 °C in spring 2012. Moisture content of these samples was compared with that of the kiln-dried samples to make sure that they were similarly dry.

For ^15^N analysis, three subsamples of 30 needles per section were drawn from the total needle harvest, after dried needles had been well mixed, resulting in a composite sample of the total needle pool of each section. These were gently washed in distilled water to remove surface residues and any residual wood dust from the processing, then redried in an 80 °C oven until mass loss had ceased (usually 24 h, although some samples remained in the oven for up to 48 h) and milled inside plastic micro test tubes in a Retsch MM400 ball mill (Retsch Ltd, Hope, UK) for 20–30 min until the sample was homogenized into a fine powder. In addition to the 13 trees sampled for ^15^N recovery within the complete needle biomass, sub-samples of three branches from each of the six vertical/horizontal combination sections for five trees were taken to separate the 2011 cohort of needles from those produced in previous years. These sections were identified by growth beyond the most recent branch whorl, and separated from the main biomass of the branch before drying. The whole yield of needles harvested from the branch for both the current-year biomass and the older biomass was weighed and dried independently to allow a calculation of the proportion of current-year needles in the section.

Sampling of the woody biomass component was performed on five trees after the needles had been removed. Cuttings were taken from the branches in each section and replicated by sampling from three entire harvested branches, distinct from the tree stem at their base, in each section. The entire branches were not homogenized for sampling but sections for analysis were taken from a range of distances along the branch to attempt to sample a representative range of tissues, taking three branch ‘cookies’ per branch per sample, containing the entire radial section 1 cm in length. These samples were washed and redried like the needles, then milled in large metal cups with two large ball bearings in the MM400 ball mill although some larger sections were split and only a radial fraction of the disc analysed. Care was taken to clean the cups thoroughly with distilled water and 100% ethanol between successive measurements. For both the needles and the branches, 2.5–3.5 mg of the milled powder per sample was weighed into a 8.5 mm ultra-clean tin capsule and analysed for [N] and δ^15^N on a SerCon Callisto CF-IRMS Isotope Ratio Mass Spectrometer (School of Biological Sciences, University of Aberdeen, Aberdeen, UK), along with standards of known isotopic abundance every 10 samples to allow the entire run to be corrected for drift. A small number (5%) of less enriched samples were analysed at the School of Geosciences, University of Edinburgh on a PrismIII dual-inlet isotope ratio mass spectrometer (VG Isotech, Middlewich, UK) with a NA2500 Elemental Analyser (CE Instruments, Wigan, UK), with some samples run on both devices to ensure comparability. When analysing particularly highly enriched samples (with δ^15^N in the 1000 s), a minor enhancement of the ^15^N ratio of subsequent samples is observed (A. Midwood, personal communication). In order to reduce the effect of this artefact, samples of suspected high enrichment were run on the mass spectrometer in order of expected increasing enrichment.

### Simple predictive model

A simple predictive model was used to calculate the expected ^15^N abundance based on the tree and canopy size. We used measurements of total dry needle biomass made at felling, as well as DBH and measured tree height (length of intact stem + stump after felling), and used allometric equations to predict the ^15^N recovery within the tree. To calculate the branch biomass of the trees we used equations for foliar and crown biomass, but as our trees had comparatively more lateral biomass than typical due to their edge profile, we used the actual needle biomass to derive crown and branch biomass by rearranging the standard equations:

(1) $$\text{DBH}=\sqrt[{p}]{\frac{\log\beta (1-\text{Needle Biomass}/\alpha_{\mathrm{needles}})-\alpha_{\mathrm{branches}}}{\gamma }}$$

as given in McKay (2003), where *α*_needles_ and *β* are constants for leaf biomass models for spruces and firs, and *α*_branches_, *p* and *γ* are species-specific constants for a crown biomass model for Sitka spruce. Branch biomass was then calculated as the difference between the crown biomass model (*α + γ *⋅* *DBH* *⋅* p* (McKay 2003)) and the measured needle biomass.

Predicted N recovery was based on biomass and measured average N%, assuming that all N in the canopy was a valid sink for the injected N, with no losses such as gaseous N emissions or leakages from the apparatus. ^15^N was allocated evenly based on the calculated size of N pools in the canopy, divided into separate branch and needle pools. No spatial variation in allocation due to radial or vertical components was included in this null model, and no enrichment was allocated to the roots, but this was assumed to be minimal (<5%) based on earlier work on Sitka spruce saplings (Weatherall et al. 2006), nor was any ^15^N allocated to stemwood, where C : N ratios are higher (Gundersen 1998), and a greater proportion of the total biomass is not growing. This assumed no net growth over the injection period (i.e., that the size of the N pools within the tree was the same at the time injected as when felled) and no losses of ^15^N due to senescence or shedding of needles from the oldest age classes of needles. While both growth and litterfall would have been ongoing in the trees, the end of the growing season is usually a period of fine root growth and starch production, rather than stem elongation (Ford and Deans 1977, Weinstein et al. 1991), and litterfall does not appear to have a seasonal component in Sitka spruce (Hansen et al. 2009).

Predicted ^15^N recovery in each section was therefore calculated as follows:

(2) $${}^{15}{\text{N}}_{\mathrm{observed}}={}^{15}{\text{N}}_{\mathrm{initial}}+{}^{15}{\text{N}}_{\mathrm{injected}}\;\left(\frac{{\text{N}}_{\mathrm{section}}}{{\text{N}}_{\mathrm{crown}}}\right),$$

where ^15^N_initial_ is the initial total ^15^N content of the section in question, N_section_ is the total N of the section (determined post-harvest based on per-section average [N]), N_crown_ is the total tree-level N specific to each individual tree and ^15^N_injected_ is the (constant) total ^15^N of the injection.

### Expressions of ^15^N recovery

The predicted and observed ^15^N atom% (referred to as ^15^N enrichment) were expected to vary among trees because of variable dilution due to tree size. Therefore we also calculated a percentage recovery (referred to as ^15^N recovery, Eq. (3)), assuming an even distribution of all injected ^15^N throughout the canopy (Eq. (2)), which allowed comparison of relative ^15^N recovery between different sections of the canopy while accounting for an expected lower ^15^N enrichment in larger trees due to dilution.

(3) $${\text{Rec}}_{\mathrm{section}}={\text{Rec}}_{\mathrm{crown}}{}^{15}{\text{N}}_{\mathrm{injected}}\left(\frac{{\text{N}}_{\mathrm{section}}}{{\text{N}}_{\mathrm{crown}}}\right)$$

with Rec_crown_ being the total recovery of the injection, in % units, specific to each tree.

### Statistical analyses

All statistical analyses were conducted in R (R Development Core Team 2008) v3.1.0.

We used analysis of variance to compare ^15^N recovery and amount of label among the trees, and examine the relationships between both of these measures of ^15^N distribution and tree-level variables such as uptake rate or tree biomass.

Among canopy sections, we constructed linear mixed-effect models to predict needle ^15^N atom%, ^15^N recovery, distribution of needle biomass and proportion of current-year needles. The triplicate samples from each section were averaged to give a single ^15^N value for each metric per section. The models all used tree as a random (block) factor, and vertical and horizontal section positions and average section-level needle biomass and N content as fixed factors. We also included two tree-level metrics as fixed factors: the ratio of canopy (needle and branch) biomass to total biomass (referred to as canopy ratio), and the total biomass of the tree. These were transformed for normal distribution if appropriate and spatial autocorrelation of ^15^N recovery among sections based on proximity within the canopy was accounted for by including a correlation matrix based on the Manhattan distance between the average modelled distal end position of all branches within each section, using the tree height, DBH and Pythagoras theorem.

We compared models with up to five-way interactions using ΔAICc (small-sample corrected Akaike Information Criterion) and dropped terms stepwise to minimize AICc until the model with the lowest AICc was found. *R*^2^ values are reported as marginal ($R_{\left(\text{m}\right)}^{2}$), indicating the proportion of variance accounted for by the fixed factors using the methodology for pseudo-*R*^2^ for mixed-effect models detailed by Nakagawa and Schielzeth (2013). Models for tree-level responses were linear regressions without the tree-level random effect, and presented as adjusted $R_{\left(\mathrm{adj}\right)}^{2}$. Branch ^15^N was compared in the same manner, but separately, due to the limited number of replicated trees. Likewise, as we only measured the proportion of current-year cohorts across six of the 13 trees, these were not included in the overall model and were analysed separately.

## Results

### Solution uptake

No damage or phytotoxic foliar ‘burns’ were observed in preliminary unlabelled tests. The 21 g l^−1^ solution took between 2 and 10 days to reach the threshold estimated concentration of 1 g l^−1^, and uptake times (mean 6.4 ± 2.3 (SD) days) displayed by individual trees were not related to total tree mass (*P* > 0.05), needle mass (*P* > 0.05) or canopy ratio (*P* > 0.05).

### Biomass harvest

**Figure 1.**
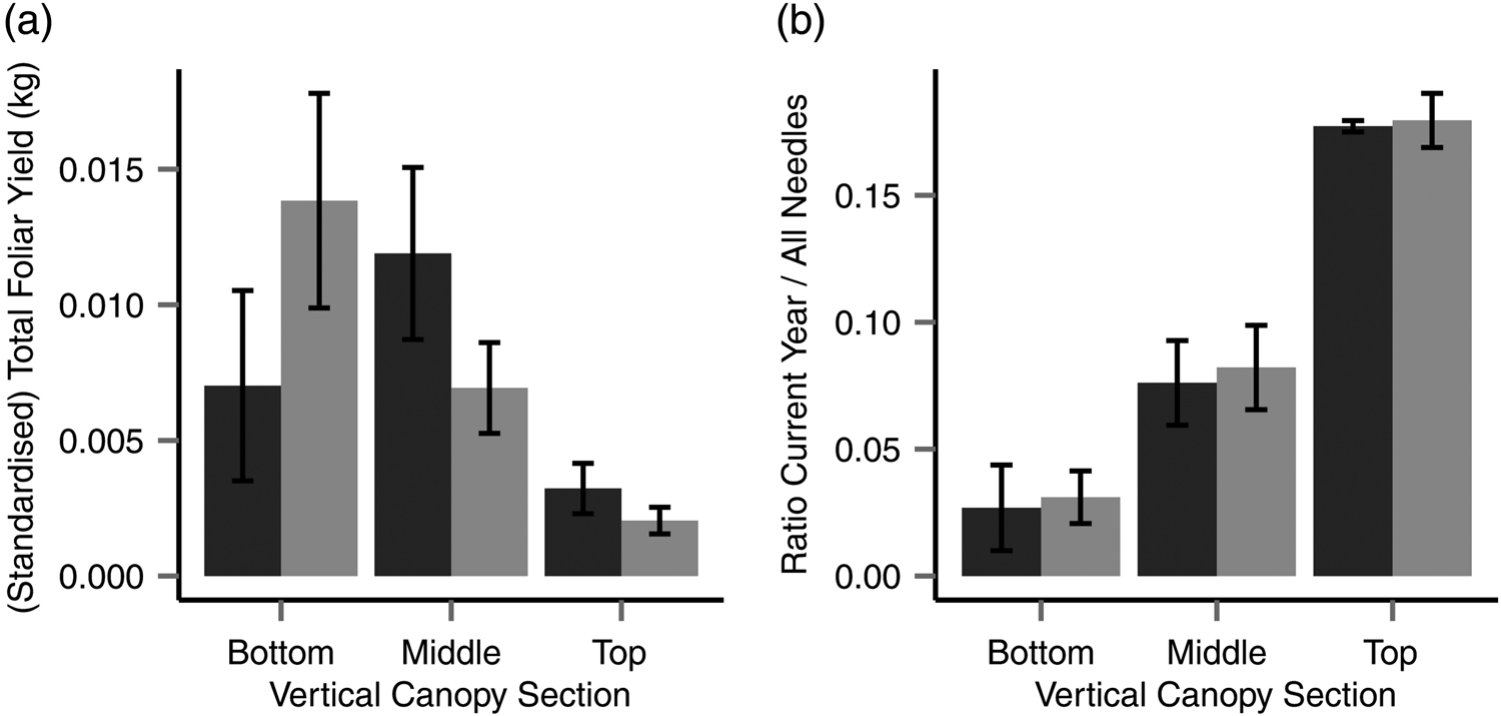
Comparison across vertical canopy sections of standardized needle biomass yield (a) and proportion of current cohort needles (b). Shading indicates lateral sections: inside the stand (dark) and outside the stand (light). Error bars show 95% CI.

At harvest in December 2011, 22.6 ± 7.3 (SD) kg needle litter was rendered per tree (293.6 kg in total). The harvested needle biomass decreased up the tree as successive sections were smaller, and was broadly evenly distributed laterally (67.3% of the mass of harvested needles were from Canopy_OUT_, two-thirds of the total circumference of the stem). When harvests were standardized using the total circumference of the tree (Figure 1a) to compare yields from an equal area, the significant variables affecting section-level needle biomass were vertical position (*P* < 0.0001), the interaction between vertical and horizontal positions and total tree height (*P* < 0.0001, $R_{\left(\text{m}\right)}^{2}$ = 0.53) but not the horizontal position (*P* > 0.05).

The fraction of the needle biomass harvested in the current-year cohort (Figure 1b, Table 2) increased vertically (Canopy_TOP_ 17.8% (CV = 4.7%); Canopy_MID_ 7% (CV = 7%); Canopy_BOT_ 2.9% (CV = 0.03%), *P* < 0.001, $R_{\left(\text{m}\right)}^{2}$ = 0.96) across the subsample (n = 6 trees), but this did not change significantly between horizontal sections (*P* > 0.05), nor was there any interaction (*P* > 0.05) between the sections.

**Table 2.** Average biomass, ^15^N abundance and proportion of current-year needles among canopy sections. Shown as mean ± SE.

Canopy position		Outside stand π/2 rad	Inside stand 3π/2 rad
Canopy_BOT_ (<3.5 m)	Needle biomass (kg)	119.92 ± 4.85	30.40 ± 2.15
	Atom% ^15^N	1.15 ± 1.24	1.42 ± 1.45
	Current-year needles (%)	2.59 ± 1.9	2.99 ± 1.1
Canopy_MID_ (3.5–7.5 m)	Needle biomass (kg)	60.07 ± 2.94	51.53 ± 1.95
	Atom% ^15^N	2.35 ± 0.70	2.23 ± 0.81
	Current-year needles (%)	7.04 ± 1.6	7.57 ± 1.6
Canopy_TOP_ (>7.5 m)	Needle biomass (kg)	17.71 ± 6.05	13.98 ± 5.69
	Atom% ^15^N	2.29 ± 0.79	2.28 ± 0.74
	Current-year needles (%)	15.0 ± 0.01	15.2 ± 0.01

### ^15^N abundance and label recovery

**Figure 2.**
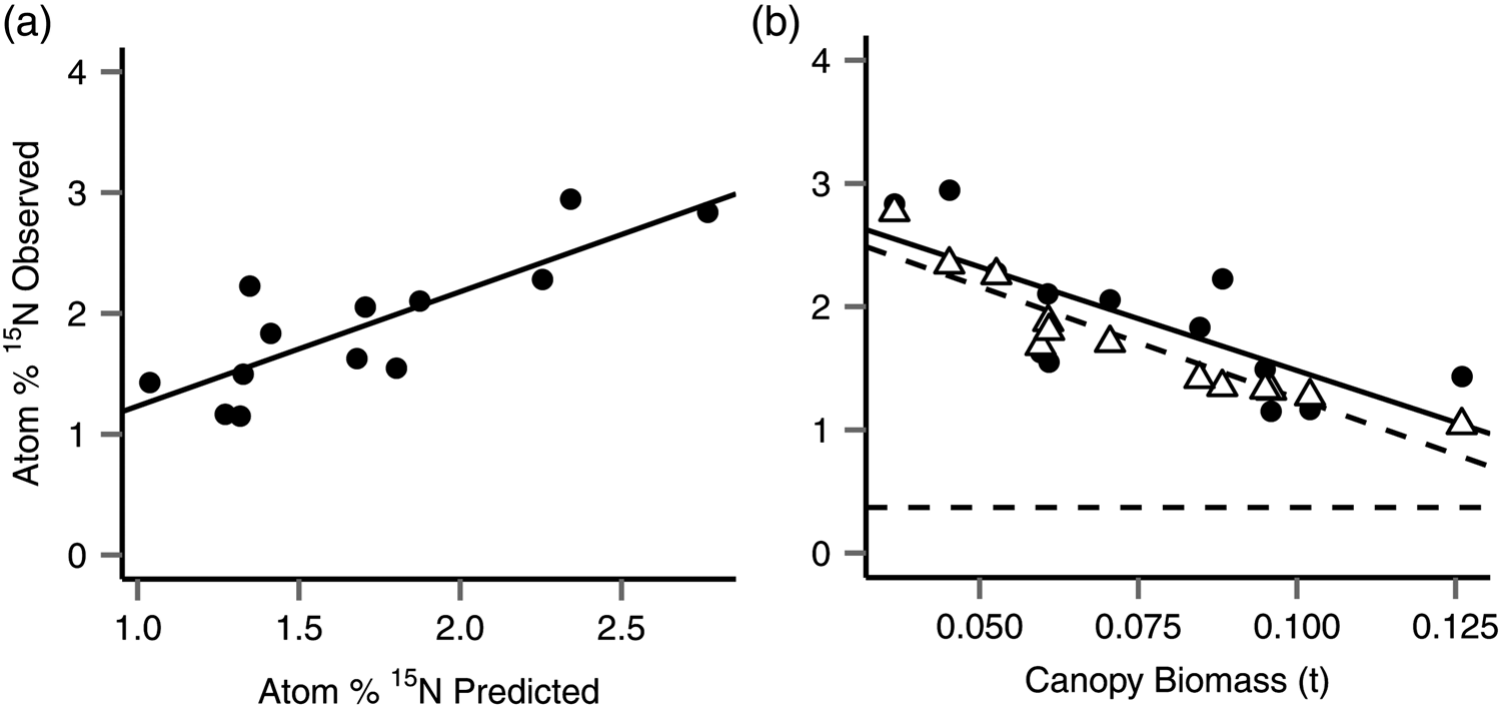
Measured ^15^N abundance was closely correlated with predicted values. (a) Correlation with linear relationship (adjusted *R*_2_ = 0.651). In (b), observed (black circles) and predicted (open triangles) needle atom% show the predicted dilution effect caused by increasing biomass. Best fit lines indicate linear relationships for observed atom% (solid) and predicted atom% (dashed), while the horizontal line indicates natural abundance.

Average per-tree needle nitrogen content was 1.18% (CV = 11%), and the average abundance of ^15^N was 1.89 atom% (CV = 30%). The baseline value of atom% ^15^N across tree sections was ∼0.38 atom% ^15^N. ^15^N abundance in the branches was 2.35 atom% (CV = 99%), while N content of the branch sections analysed was 0.6% (CV = 44%). The observed needle ^15^N atom% when considered on the level of individual tree crowns correlated with the prediction of Eq. (2) (*P* < 0.001, $R_{\left(\text{adj}\right)}^{2}$ = 0.651, Figure 2a), decreasing with increasing canopy biomass (*P* = 0.003, Figure 2b), and with canopy ratio (*P* = 0.025), with $R_{\left(\text{adj}\right)}^{2}$ = 0.571, but was not related to total biomass (*P* > 0.05), N contents of needles (*P* > 0.05) or ranked uptake rate of solution (*P* > 0.05). Branch ^15^N abundance also broadly correlated with predicted ^15^N recovery, based on the estimated branch biomass (Eq. (1), *P* = 0.039, $R_{\left(\text{adj}\right)}^{2}$ = 0.587).

This ^15^N abundance meant that an average of 53.1% (CV = 29%) of the total ^15^N injected into the stem was accountable in the needles, and an average of 68.5% (CV = 81%) was accountable in the branches, totalling 118.4% (CV = 43%) of the total ^15^N injected. In the needles, 112.9% (CV = 20%) of the predicted ^15^N recovery was found, while 89% (CV = 73.7%) was found in the branches. There was no effect of canopy (*P* > 0.05) or tree size, ranked uptake rate (*P* > 0.05) or average needle % N content (*P* > 0.05) on the recovery of the total injection returned in the needles (*P* > 0.05) or branches (*P* > 0.05) when totalled for the tree. ^15^N recovery was highly variable among trees with a minimum of 33.5% of the injection returned in foliage, a maximum of 88.9% and a mean of 53.1% (CV = 28%).

**Figure 3.**
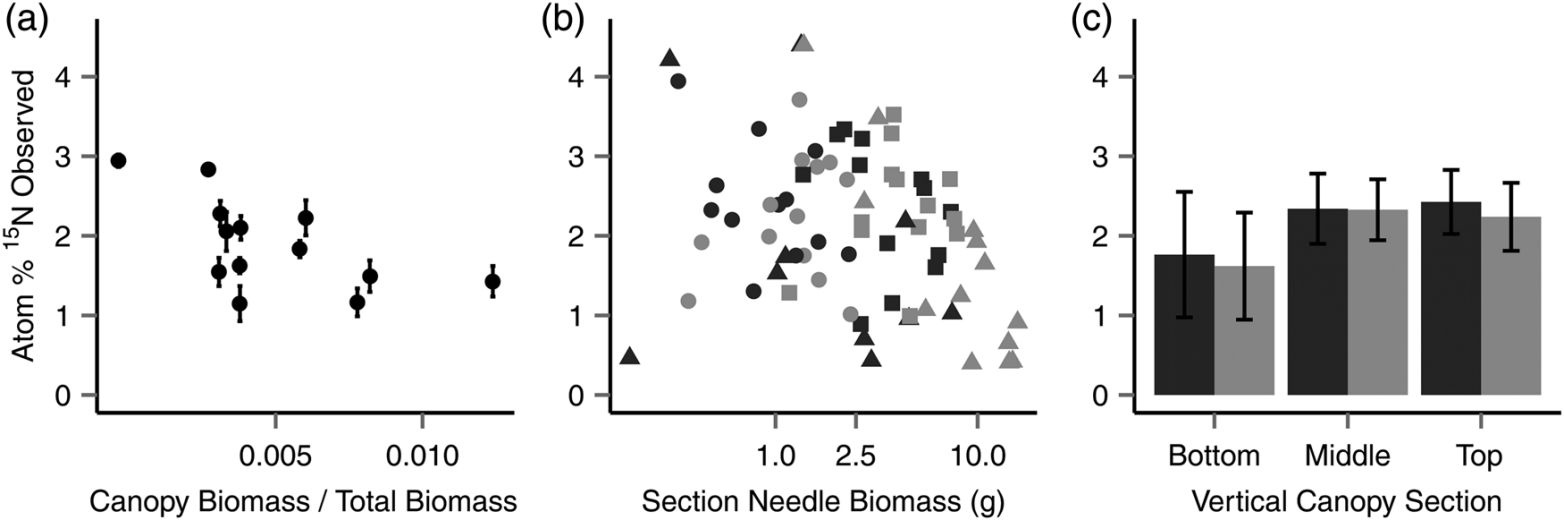
Relationship between atom% ^15^N measured in the needle biomass of (a) the entire trees and (b and c) of individual tree sections, compared with (a) the ratio of whole canopy/tree biomass, (b) the harvested needle biomass, (c) canopy section. In (b) and (c), lateral canopy sections are shaded dark grey (inside the stand) and light grey (outside the stand), and in (b), canopy sections are divided into Canopy_BOT_ (triangle), Canopy_MID_ (square) and Canopy_TOP_ (circle). Error bars show 95% CI.

^15^N enrichment varied among the six canopy sections. Despite the lower average abundance, the bottom sections of the canopy displayed both the highest individual needle enrichment (4.39 atom%) and the lowest enrichments (0.399 atom%). ^15^N abundance in the needles was driven by vertical position (*P* = 0.016), canopy ratio (*P* = 0.004) and needle biomass (*P* = 0.0305) (Figure 3, Table 3), with a greater ^15^N enrichment displayed in smaller sections, smaller canopies and higher up the tree; Canopy_TOP_ (2.33 atom%, CV = 25%) and Canopy_MID_ (2.33 atom%, CV = 24%), were significantly (*P* < 0.05) greater than Canopy_BOT_ (1.68 atom%, CV = 101%), but not significantly different from each other (Tukey HSD, *P* = 0.451). Neither total biomass nor any interaction terms remained in the most parsimonious (AICc) model when reduced by stepwise regression, which had a $R_{\left(\text{m}\right)}^{2}$ of 0.28.

**Table 3.** Summary of most parsimonious linear models for needle ^15^N atom% and needle ^15^N recovery (as % predicted in section).

Variable	Degrees of freedom (numerator)	Degrees of freedom (denominator)	*F*-value	*P*-value
^15^N atom% model				
Intercept	1	58	449.63	<0.0001
Vertical section	2	58	4.474	0.0156
Ratio canopy/whole tree biomass	1	11	13.1145	0.004
Needle biomass in section	1	58	4.9162	0.0305
^15^N recovery (as % predicted in section) model				
Intercept	1	58	254.63674	<0.001
Vertical section	2	58	8.74377	0.0005
Needle biomass in section	1	58	3.39084	0.0707

**Figure 4.**
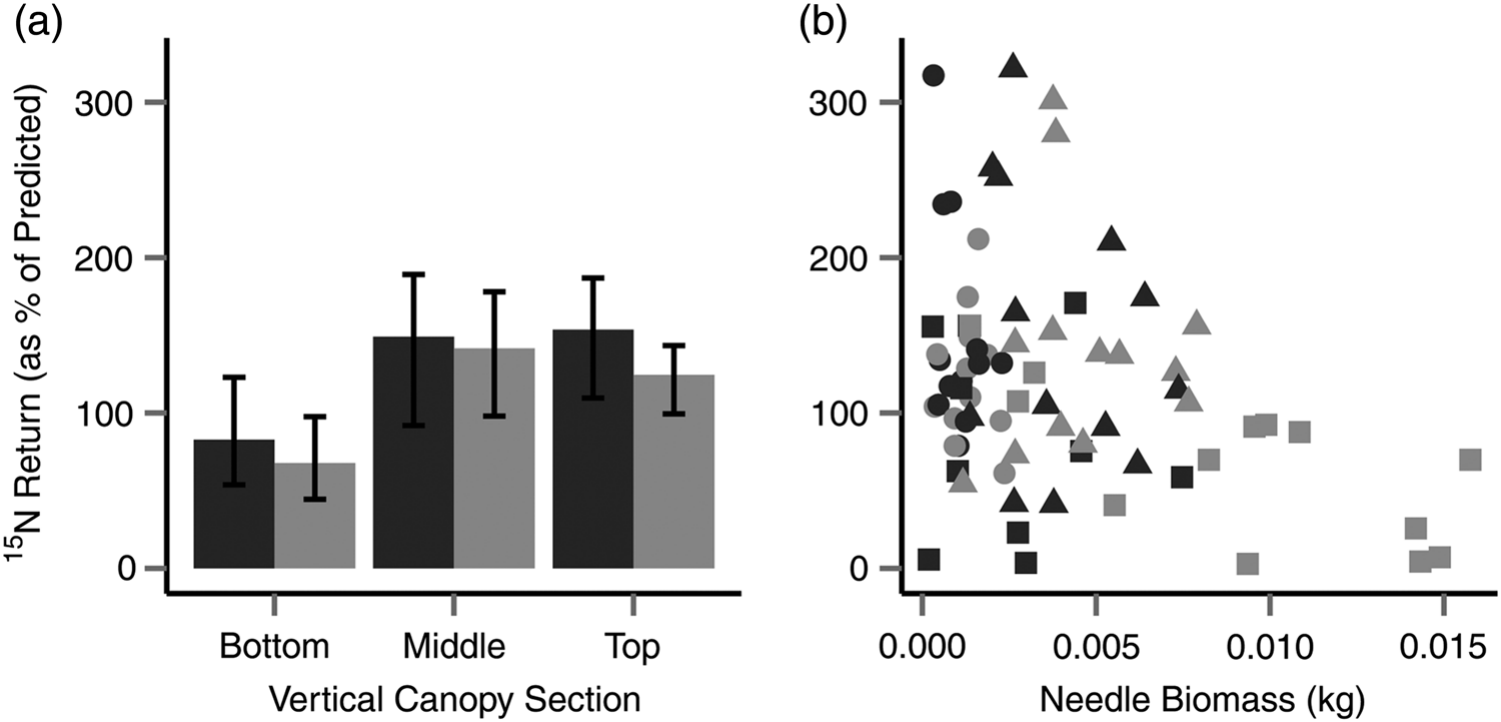
Relationship between recovery of predicted ^15^N (%) with respect to (a) vertical sections and (b) needle biomass. Lateral canopy sections are shaded dark grey (inside the stand) and light grey (outside the stand), and in (b), sections are represented as Canopy_BOT_ (triangle), Canopy_MID_ (square) and Canopy_TOP_ (circle). Bars in (a) show 95% CI.

This difference led to Canopy_BOT_ accounting for considerably less N (88.3 ± 61.8%) than Canopy_MID_ (163.8 ± 69.9%) and Canopy_TOP_ (158.4 ± 44.2%). The canopy ^15^N allocation (Table 3) was significantly related only to vertical section (*P* = 0.0005, Figure 4), although normalized needle biomass remained in the most parsimonious model (*P* = 0.0707). The $R_{\left(\text{m}\right)}^{2}$ for this model was 0.23. Among the vertical sections of the canopy (Tukey HSD), there was a significant difference in ^15^N allocation between Canopy_MID_ and Canopy_BOT_ (*P* < 0.001), but no significant differences in recovery in Canopy_TOP_ against recovery to the Canopy_MID_, or between Canopy_TOP_ and Canopy_BOT_, were found.

**Figure 5.**
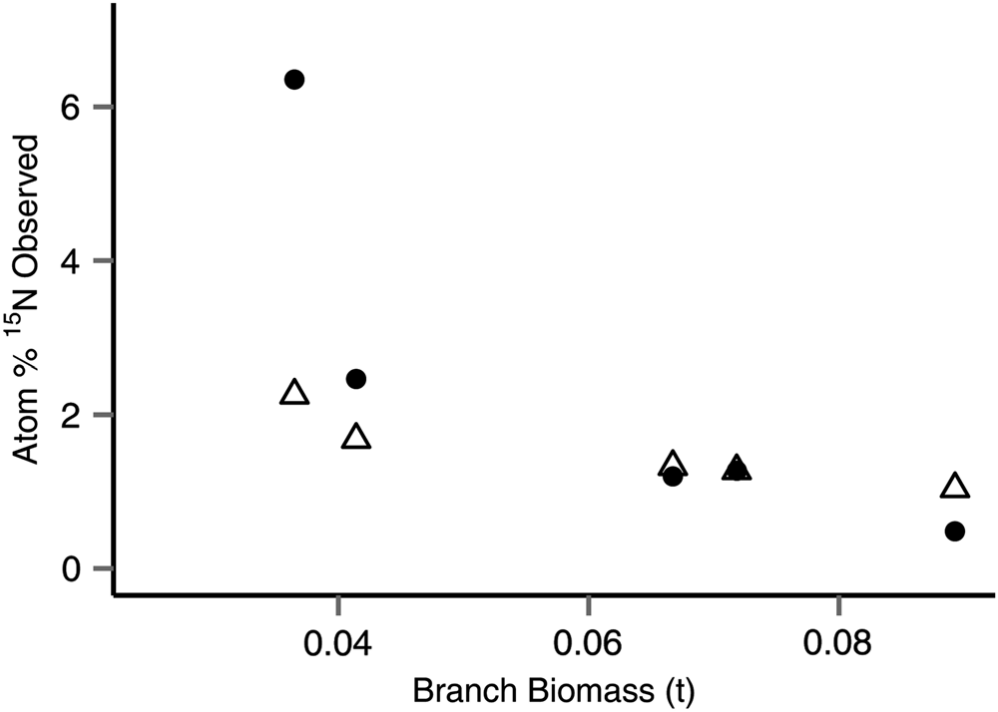
Mean observed (atom%) ^15^N label in branch sections, compared with total branch biomass for each tree. White triangles are predicted ^15^N abundance, and solid circles are observed ^15^N abundance.

Within individual trees, observed ^15^N abundance in branches was much more variable than in needles (Canopy_BOT_ 2.69 atom% (CV = 137%); Canopy_MID_ 3.45 atom% (CV = 89%); Canopy_TOP_ 2.11 atom% (CV = 69%)), driven by an apparent heterogeneity of recovery, particularly in Canopy_BOT_ where some samples displayed atom% at natural abundance while others were as high as 10.4 atom% (the highest recorded). Average atom% varied among trees and with biomass (Figure 5). When the observed atom% was expressed as a recovery of the expected label, this varied among sections but there was no significant statistical relationship with the measured variables.

## Discussion

The variability among species investigated, concentrations of solution, objectives and methods reported in the literature makes it difficult to compare results from stem injection experiments. Given the presence of multiple age cohorts of needles in evergreen conifers, variation in the expression of an isotope label would also be expected. We found a high variability in the atom% ^15^N abundance, and therefore limit discussion as far as possible to injections of conifers where the intra-canopy N dynamics are expected to be as comparable as possible to our trees.

### Overall ^15^N recovery

The harvested biomass of every injected tree was ^15^N enriched, in both the needles and the branches, with an average recovery >121% of the applied label when the measured ^15^N excess was scaled to the entire canopy. This was fairly variable (CV = 22.8%), predominantly due to the high and variable label recovery in the branches (CV = 99.7%) which contributed 46.9% of the calculated total label recovery, with the average branch atom% matching or exceeding the prediction in four of the five trees analysed despite the prediction assuming all N was assigned to branches or needles. The predictive model assumed a similar turnover rate (and therefore a similar proportion of N replaced) between foliar and branch pools based on observed branch and needle N content, but real differences in this rate may have driven the discrepancies from predictions. As there are no alternative explanations for an additional enriched ^15^N source for the trees, the high total recovery for the ^15^N label in the canopy from these measurements contrasts with Seiter and Horwath (1999) and Garten and Brice (2009), who inferred a large allocation of injected N to belowground processes from low accountancies in aboveground tissues. While the greatest foliar ^15^N recovery may be expected when injections are timed with periods of peak foliar growth, refilling of N storage pools in conifer foliage may also account for a strong ^15^N recovery in needles. This experiment took place late in the growing season, when most growth is in roots and structural tissues (Weinstein et al. 1991), and when root N uptake is greater than plant N demand (Millard and Grelet 2001). The high canopy ^15^N content observed is consistent with sequestration of this additional N in overwinter storage pools in the needles, while the belowground demand for N may be fully satisfied by ongoing root uptake.

### Needle ^15^N distribution

Overall, measured needle biomass for each section varied considerably beyond the expected allometric distribution based on tree size, presumably because of the edge nature of the trees. This accounted for 53.1% of the overall canopy ^15^N in excess of natural abundance, considerably greater than other studies using smaller trees (e.g., Horwath et al. 1992, Augusto et al. 2011), although Proe et al. (2000) found a similar recovery (45%) in the foliage of 5–8 m conifers, 1 week after injection. While it is difficult to compare label recovery between studies, our canopy estimates of ^15^N recovery are substantially greater than Augusto et al. (2011) (42–62%), which suggested that their recovery may be due to lower canopy : biomass ratios (Ritson and Sochacki 2003) in their larger trees compared with Proe et al. (2000), while we used edge-profile trees with relatively large canopies which may have contributed to their relatively greater short-term allocation to a relatively larger crown.

Our samples were all collected 4.5 months after the injection, in winter, and were from the entire foliar biomass (including both the 2011 needle cohort and older needles). At this time, needle ^15^N abundance was expected to be biased towards current-year needles (Augusto et al. 2011) as most conifers store N in roots and 1-year-old needles, in photosynthetic proteins such as RuBisCo (Millard et al. 2007), remobilizing this N in the next growing season (Millard and Proe 1992). Foliar ^15^N abundance was biased towards the upper canopy, consistent with Proe et al. (2000), where crown zones were assigned based on age of the relevant stem section. Our results are, however, in contrast to Augusto et al. (2011), who reported no difference between upper and lower canopies. Canopy nitrogen storage is thought to be more important in larger trees (Miller 1986), as they have larger pools of current-year needles available, and greater N requirements in the spring. Nitrogen storage pools can be rapidly mobilized to overcome limited uptake from the soil, providing a resource ready for the development of new foliage the next growing season (Augusto et al. 2011). Current-year needles, expected to be the store for this excess ^15^N, were ∼2.8, 7.3 and 15.1% of the total foliar yield for Canopy_BOT_, Canopy_MID_ and Canopy_TOP_, respectively, significantly biased towards the upper canopy sections.

However, ^15^N atom% abundance and recovery did not exactly follow this distribution, with equal apparent total allocation of injected ^15^N to the middle and upper foliage regardless of the difference in the total mass of new cohort needles. ^15^N labels absorbed from the soil are typically found in regions of high metabolic rates (Mead and Pritchett 1975), which are usually located within the canopy in regions of greater exposure to sunlight and more photosynthetic potential (Ellsworth and Reich 1993, Hollinger 1996), and the apparent inconsistency in N allocation compared with new needles may be due to different spatial demands for N for photosynthetic function.

Dilution (Swanston and Myrold 1998) explained much of the variation in ^15^N atom% in the most parsimonious model, both at the level of individual trees (canopy ratio) and in individual sections within the trees (needle section biomass), but when this effect was removed by the ^15^N recovery model, only vertical position of the section remained significant, average recovery in the upper canopy being greater than in the lower canopy. Respectively, the ^15^N atom% and recovery models explained 28 and 23% of the variability in the amount of ^15^N label, calculated from 30 needles per individual sample with considerable variation typically found among replicates from the same section. The within-section variability was not explainable by the measured variables as, aside from N content of samples, no explanatory variables were measurable to the individual sample level. Exposure (Kohyama 1980, Zavitkovski 1981) and competition (Vanninen and Mäkelä 2000) would have varied within each canopy section due to individual needle positions, as well as differing amounts of age classes (Norman and Jarvis 1974). Alternatively, uneven allocation may have been due to the heterogeneous distribution of the label within the tree over the time period of the study.

### Branch ^15^N distribution and contrast with foliar ^15^N

Branch atom% ^15^N was even more varied than in needles, with recorded atom% as high as 10.66% but often with measured ^15^N at natural abundance levels, especially so in Canopy_BOT_, where the coefficient of variation was 137%. This variance was mostly due to one of the five trees analysed for branch ^15^N having a consistently very high ^15^N enrichment in the branches (resulting in an average branch atom% in the whole tree of > 6%), with it having the third highest needle ^15^N content of the 13 trees, and highest from the five trees where branches were also analysed.

Wood contained a much larger range of ages and potentially a greater range of living tissues within individual branches, especially in larger trees where needle lifespan is much shorter than the age of the tree. Depending on the position of the branch, there was also potential variability in growth and metabolic rate among branches due to environmental factors. We also used the needle biomass to predict the branch biomass in the allometric model, rather than direct measurement, expecting it to be more accurate than DBH for these trees where release from competition would increase branchiness (Mäkinen and Colin 1998, Ackerman et al. 2013). If, in this case, we substantially overestimated the branch masses, this would also have caused an overestimation in the label recovery in the branches.

Alternatively, this high variation in both ^15^N recovery and ^15^N atom% (which we measured directly and is not dependent on branch biomass estimates) may also have been due to variation in N allocation. Sap flow in many trees is sectorial (Larson et al. 1994, Orians et al. 2004, Gloser et al. 2008), and the injection in summer 2011 may have initially reached specific regions of the canopy in the same sector as the injection site. As foliar N pools are dynamic and N is assigned both to maximize photosynthetic capacity across the canopy and for storage (independent of plant N (Millard and Grelet 2001, Fife et al. 2008)), variation in needle and branch ^15^N may have been due to a more gradual movement of ^15^N to some parts of the canopy.

In the autumn, N uptake is typically greater than total tree N demand, as shoot extension and foliar production have ceased (Weinstein et al. 1991), but production of storage proteins continues. In contrast, no major N storage in conifers occurs in bark and wood (Millard and Grelet 2001) during this period, and radial wood production in branches, stems and roots is ongoing in early autumn (Weinstein et al. 1991). New wood laid down following the August injections may be a more continuous structural sink for ^15^N, while foliar sinks may be more transient as the N moves around the canopy in order to maximize ^15^N storage in foliage at the end of the year. The high branch ^15^N in some branches may reflect the branches first reached by the ^15^N label and the highest ^15^N abundances in branches at the base of the canopy may be structural sinks closest to the injection site where the additional ^15^N is least diluted by N already in the sap, translocated from foliage throughout the upper canopy.

Rates of uptake of the solution from the injection site varied among trees but did not correlate with ^15^N abundance or recovery of the expected label, and there were no relationships between the uptake rates and measured total biomass, canopy size or ratio, needle biomass or total N% (all *P* > 0.05). In non-labelled experiments, these rates are highly variable (Sánchez-Zamora and Fernández-Escobar 2004) among tree species and seasons of injection. While we expected uptake to be rapid due to movement of the xylem stream (Meinzer et al. 2001), this variation may have been due to the difficulty of standardizing stem wounding, and accessing different depths of the stem with different flow rates (Delzon et al. 2004). Variations in canopy morphology (Fiora and Cescatti 2008) within individual trees may also have driven differences in relative flow rate experienced by a single location radially, or around the circumference (Čermák et al. 2007) of the stem.

There was no significant difference in total needle biomass between the inside and the outside of the stem, once this was adjusted to compare identical proportions of the circumference despite expectations due to the trees' position on the edge of the stand and a well-known release from competition on the exposed size. As branch biomass was calculated using an assumed linear relationship with measured needle biomass, the very high recovery in some sections may be a result of this relationship varying throughout the canopy.

## Conclusion

We were able to successfully label the entire canopy with an apparent total recovery of the label in both the needles and branches based on scaling the ^15^N recovery through the canopy biomass. The entire needle biomass was the main sink for the injected ^15^N, accounting for over 50% of the total injection, allocation being greatest towards the upper canopy, which contains a greater proportion of young needles. ^15^N recovery in branch biomass was considerably more varied, particularly at the bottom of the tree, likely due to the distribution of sap flow and the demand for N for growth in wood, but not foliage, during the autumn. The overall high recovery can partly be attributed to the habit of the trees and the method of injection, which is well established to allow higher recovery of applied ^15^N than soil applications, but it is likely that the seasonality of the injection also played a part in the variation observed as at other times of the year N may be assigned in different proportions to above- and belowground pools due to phenological growth patterns. These differences highlight the importance of considering seasonal N dynamics and partitioning of the ^15^N label among biomass age classes in stem injection studies, particularly in conifers, while overall it is clear that the technique is a viable and efficient method for creating ^15^N biomass labelled in a cheaper way, and on a larger scale than using a labelled fertilizer on saplings.

## Conflict of interest

None declared.

## Funding

This work was funded by the UK Natural Environment Research Council (NERC), grant NE/G00725 X/1 and Forestry Commission UK. Funding to pay the Open Access publication charges for this article was provided by the UK Natural Environment Research Council.
